# Activity-based costing in services: literature bibliometric review

**DOI:** 10.1186/2193-1801-2-80

**Published:** 2013-03-05

**Authors:** Nara Medianeira Stefano, Nelson Casarotto Filho

**Affiliations:** Program in Production Engineering, Federal University of Santa Catarina, Florianopolis, Santa Catarina Brazil

**Keywords:** Bibliometrics, Bibliography portfolio, Costs, Services, Journals, 00–02

## Abstract

This article is aimed at structuring a bibliography portfolio to treat the application of the ABC method in service and contribute to discussions within the scientific community. The methodology followed a three-stage procedure: Planning, execution and Synthesis. Also, the process ProKnow-C (Knowledge Process Development - Constructivist) was used in the execution stage. International databases were used to collect information (ISI Web of Knowledge and Scopus). As a result, we obtained a bibliography portfolio of 21 articles (with scientific recognition) dealing with the proposed theme.

## Introduction

Managers need certain information to improve the efficiency of their management. They also lack answers to two very important questions: what are the sources of profitability, and how can the organization's performance be improved? Managers cannot make decisions without reliable information about costs, so the need to calculate product costs or services through the Activity-Based Costing (ABC) is emphasized.

The ABC method has the fundamental characteristic to seek to reduce distortions caused by arbitrary allotment of indirect costs acquired in traditional systems. Indeed, ABC is one of the methods made and published about the application of this method (Gunasekaran et al., 1999; Hussain and Guanasekaran 2001; Cotton et al., 2003; Kellermanns and Islam, 2004; Kallunki and Silvola, 2008; Duh et al., 2009; Dugel and Bianchini, 2011; Stefano, 2011; Lutilsky and Dragija, 2012; Jänkälä and Silvola, 2012; Schulze et al., 2012).

The ABC approach treats the client as the object of cost analysis, in parallel with the analysis of ownership costs for suppliers (Niraj et al., 2001; Narayanan and Sarkar, 2002; Anderson, 2005; Salem-Mhamdia and Ghadhab 2012). The emphasis is on getting a better understanding of the behavior of indirect costs. The ABC system is designed and implemented on the premise that products consume activities, activities consume resources and resources consume costs. Thus, the terms activities, drivers and resources are important for understanding ABC.

An activity is the result of the combination of technological and financial material and human resources used to produce goods and services. The cost driver is the way in which costs are assigned to activities, they form the basis of ABC, and seek to trace the origin of the cost and establish a relationship of cause/effect (Stefano et al., 2010). Resources or inputs are necessary expenses, arising from regular operations of the organization, such as depreciation, water, wages and electricity. The amount of each driver that is associated with the activity that you want to cover is called a factor of resource consumption.

With economic development and increased competitiveness, the services sector began to look for new concepts in management, so it could monitor the market, increasingly demanding. Despite the different characteristics in relation to the manufacturing (Gunasekaran and Sarhadi, 1998) sector, it has been seeking and adapting concepts used successfully in the cost area.

The current economic climate meant that the service organizations feel the need to know, control and manage their costs effectively. Hence, the importance of investing in programs aimed at reducing production costs. Expenses that with some care, could often be easily prevented or at least reduced, often turn out to link the final cost of products and/or services. In general, the cost controls in service organizations have some points in common with those practiced in the industry. Such issues are production order (Lins and Silva, 2005), contribution margin and balance point, and can be applied in many service organizations.

Therefore, within this context, the aim of this article is to structure a bibliography portfolio to check the use of the ABC method in service (Susskind, 2010; Büyüközkan et al. 2011; Büyüközkan and Çifçi 2012; Grissemann and Stokburger-Sauer, 2012; Gunasekaran and Spalanzani 2012; Calabrese, 2012) and contribute to discussions within the scientific community. The methodological approach used was a literature review based on bibliometrics (Førsund and Sarafoglou 2005; Tsai, 2011; Tan et al., 2010; Tsay and Zhu-yee 2011; Gumpenberger et al., 2012; Van Raan, 2012) and qualitative and quantitative analysis of the articles. The databases chosen to select the publications was portal ISI Web of Knowledge e Scopus for being comprehensive and multidisciplinary (and can be accessed via the portal Capes), and period of searches comprises 1990–2011. The methodology followed a three-stage procedure: planning, execution and reporting. Process *ProKnow*-C (*Knowledge Process Development - Constructivist*) was also used in the stage of execution.

Besides this introduction, the paper presents: (i) research methodology, (ii) results; (iii) the results and (iv) final considerations; and, finally, (v) references used.

## Research methodology

This section discusses choice procedures and methodology description.

## Methodology choice

An analytical review is necessary to systematically assess the contribution of a particular literature topic. Generally, the review process consists of three parts: data collection, data analysis and data synthesis. The scientific rigor in the conduct of each of these steps is critical to an analysis of its quality. Data collection can be done in different ways. As an example, using existing knowledge in the literature to select articles and search various databases using keywords.

Once items are selected for review, data analysis can proceed in different ways, depending on the objectives of the revision (Crossan and Apaydin, 2010). A review to consolidate the results of several empirical studies may depend on either qualitative or quantitative analysis of the results. Data synthesis is the main product of the research as it produces new knowledge based on complete data collection.

This research is exploratory and descriptive (Richardson 2008). It is exploratory because it follows a process to build a bibliographic portfolio of articles in a given topic. It is descriptive because it seeks to describe the characteristics of scientific publications of this portfolio and its references, in case, the application of ABC method of costs in services.

## Methodology description

**Figure 1:**
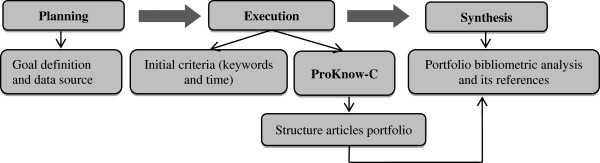
**Proposed structure for the paper.** Source: Authors.

For this paper a three-stage procedure was followed: Planning, Implementation and Synthesis (Figure 1). During the planning phase, the research objectives were defined and the sources of data were identified. The second stage, implementation, consists of two sub-steps: identifying the initial selection criteria (time, databases and keywords) and using the *ProKnow*-C (*Knowledge Development Process–Constructivist*).

ProKnow-C is proposed by Ensslin et al. (2010) to build knowledge based on a researcher’s interests and boundaries, according to the constructivist view. This instrument (Bortoluzzi et al., 2011), provides the steps to be followed for the construction of a Bibliographic Portfolio selection representing the topic that you want to search. This phase is divided into two steps: (a) Selection of gross articles stock (2) filtering the stock items, which is secreted into five sub-steps: (a) gross articles stock filter regarding redundancy; (b) non-recurrent gross articles stock filter regarding title alignment, (c) non-recurrent gross articles stock filter with title alignment regarding scientific recognition, and (d) article reanalysis process that do not have science recognition, (e) filter regarding complete article alignment.

The third and last step concerns the synthesis that is the portfolio bibliometric analysis and its references. It was chosen to be limited only to journals as data sources because they can be considered validated knowledge and are likely to have greater impact. Articles published in conferences and seminars were not considered, as well as books, dissertations and theses. *ISI “Web of Knowledge”* and *Scopus* databases were chosen because the databases are comprehensive and multidisciplinary. Interesting, the characteristic of these bases is that they have the scores of citations of articles, and this allows a screening of a series of articles based on this criterion.

The number of times an article is cited in Google Scholar was considered, for in ISI Web of Knowledge and Scopus only the journals or databases within it counted and not all the bases where this article is. For example, the article Improving hospital cost accounting with activity-based costing on 13/03/2012, shows that its citation number is 59, in Scopus. Now, when we use the same article and check it on *Google Scholar*, the number of citations shown is 119, this means that all bases where this article appears are counted. The time used for the search was 1990 to 2011.

## Results

Section 2 of the work presents the analysis relating to research data.

### Portfolio building

**Figure 2:**
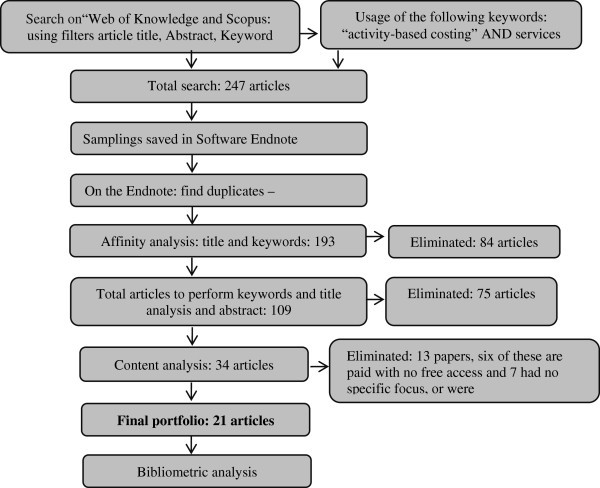
**Results of the steps of building an article portfolio.** Source: Authors.

The phase selection of gross articles stock was completed with 247 articles found (Figure 2), according to the search criteria provided.

For the next step, article stock filtration was performed, then stored in the management software bibliographic references *EndNote version Web*. This second stage was divided into three separation stages as to: (i) title alignment (ii) scientific recognition (citation number) of the articles and abstracts reading and, (iii) complete reading of the articles. Figure 2 shows a summary of the steps.

The review stage was performed with 34 articles, with proven scientific recognition, from these: 13 were eliminated (six bases were paid without access via portal Capes, and 7 had no specific focus of ABC method of costs in services, because they addressed only services or only ABC). At the end, there were 21 remaining articles, which build the portfolio on the topic in question (Table 1).

**Table 1:** **Bibliographic portfolio of articles**

Title	Authors	***Journal***	Years
A generalised cost-estimation model for job shop	Aderoba, A.	International Journal of Production Economics	1997
Application of activity-based costing to a land transportation company: a case study	Baykasoǧ lu, A.; Kaplanoǧ lu, V.	International Journal of Production Economics	2008
Applying activity based costing model on cost accounting of provider of universal postal services in developing countries	Blagojević, M.; Marković, D.; Kujačić, M.; Dobrodolac, M.	African Journal of Business Management	2010
Improving hospital cost accounting with activity-based costing	Chan, Y. C.	Health Care Management Review	1993
Costing police services: the politicization of accounting	Collier, P. M.	Critical Perspectives on Accounting	2006
Customer profitability analysis with time-driven activity-based costing: a case study in a hotel	Dalci, I.; Tanis, V.; Kosan, L.	International Journal of Contemporary Hospitality Management	2010
Health-care financial management in a changing environment	Devine, K.; O'clock, P.; Lyons, D.	Journal of Business Research	2000
Development of an activity-based costing model to evaluate physician office practice profitability	Dugel, P. U.; Tong, K. B.	Ophthalmology	2011
A new method of accurately identifying costs of individual patients in intensive care: the initial results	Edbrooke, D. L. Stevens, V. G.; Hibbert, C. L.;	Intensive Care Medicine	1997
Activity-based costing in user services of an academic library	Ellis-Newman, J.	The Journal of Academic Librarianship	2003
The cost of library services: Activity-based costing in an Australian academic library	Ellis-Newman, J.; Robinson, P.	Library Trends	1998
Providing professional mammography services: financial analysis	Enzmann, D. R.; Anglada, P. M.; Haviley, C.; Venta, L. A.	Radiology	2001
Building an activity-based costing hospital model using quality function deployment and benchmarking	González, M. E.; Quesada, G.; Mack, R.; Urritia, I.	Benchmarking: An International Journal	2005
Management accounting systems in Finnish service firms	Hussain, M. M.; Gunasekaran, A.; Laitinen, E. K.	Technovation	1998
The application of activity-based costing in the United Kingdom's largest financial institutions	Innes, J.; Mitchell, F.	The Service Industries Journal	1997
Time-driven activity-based costing for inter-library services: a case study in a university	Pernot, E.; Roodhooft, F.; Van den Abbeele, A.	The Journal of Academic Librarianship	2007
Activity-based costs of blood transfusions in surgical patients at four hospitals	Shander, A.; Hofmann, A.; Ozawa, S.; Theusinger, O. M.; Gombotz, H.; Spahn, D. R.	Transfusion	2010
Costs and effects in lumbar spinal fusion: a follow-up study in 136 consecutive patients with chronic low back pain	Soegaard, R.; Christensen, F. B.; Christiansen, T.; Bünger, C.	European Spine Journal	2007
Logistic costs case study: an ABC approach	Themido, I.; Arantes, A.; Fernandes C;. Guedes A P.	Journal of the Operational Research Society	2000
Activity-based management and traditional costing in tourist enterprises (a hotel implementation model)	Vazakidis, A.; Karagiannis, I.	Operational Research	2011
Application of activity-based costing (ABC) for a Peruvian NGO healthcare provider	Waters, H.; Abdallah, H.; Santillán, D.	International Journal of Health Planning and Management	2001

Source: Authors.

In the next subsection, the results of the portfolio profile will be presented, constructed as: recognition of scientific articles, journals that most stand out; featured authors and keywords used.

### Bibliographic portfolio of articles analysis

Performed the entire procedure to build a representative bibliographic portfolio to discuss the use of the ABC method in services, the next step was to treat these articles through bibliometric analysis.

Bibliometrics (Dorban and Vandevenne, 1992; Macias-Chapula, 1998; Mukherjee, 2009; Tasca et al., 2010) is a technique that allows situating the research through various indicators and relationships. As the indicators can be used, the number of citations, co-authorship, number of patents, as well as maps can be made of scientific fields and countries. Table 2 shows the scientific recognition indicator held in the article portfolio.

**Table 2:** **Number of citations (scientific recognition) of the bibliographic portfolio**

Title	Number of citation
Improving hospital cost accounting with activity-based costing	119
A new method of accurately identifying costs of individual patients in intensive care: the initial results	75
Activity-based costs of blood transfusions in surgical patients at four hospitals	46
The application of activity-based costing in the United Kingdom's largest financial institutions	44
Logistic costs case study - an ABC approach	35
A generalised cost-estimation model for job shop	33
Management accounting systems in Finnish service firms	29
Application of activity-based costing to a land transportation company: a case study	27
Providing professional mammography services: financial analysis	27
The cost of library services: Activity-based costing in an Australian academic library	23
Costing police services: the politicization of accounting	21
Application of activity-based costing (ABC) for a Peruvian NGO healthcare provider	21
Building an activity-based costing hospital model using quality function deployment and benchmarking	18
Time-driven activity-based costing for inter-library services: a case study in a university	19
Activity-based costing in user services of an academic library	18
Health-care financial management in a changing environment	16
Costs and effects in lumbar spinal fusion: a follow-up study in 136 consecutive patients with chronic low back pain	15
Customer profitability analysis with time-driven activity-based costing: a case study in a hotel	5
Development of an activity-based costing model to evaluate physician office practice profitability	4
Activity-based management and traditional costing in tourist enterprises (a hotel implementation model)	2
Applying activity based costing model on cost accounting of provider of universal postal services in developing countries	1

Source: Authors.

Table 3 shows the indicator journals titles (Hassini et al., 2012) presents in articles of the bibliographic portfolio.

**Table 3:** **Importance of*Journals*in the bibliographic portfolio**

Journal	Numbers of articles
International Journal of Production Economics	2
The Journal of Academic Librarianship	2
African Journal of Business Management	1
Benchmarking: An International Journal	1
Critical Perspectives on Accounting	1
European Spine Journal	1
Health Care Management Review	1
Intensive Care Medicine	1
International Journal of Contemporary Hospitality Management	1
International Journal of Health Planning and Management	1
Journal of Business Research	1
Journal of the Operational Research Society	1
Library Trends	1
Operational Research	1
Ophthalmology	1
Radiology	1
The Service Industries Journal	1
Technovation	1
Transfusion	1

Source: Authors.

In Table 3, one can find that the journals are of different areas, i.e., the ABC method of cost is applied to different types of services, whether health, libraries, transportation, security, financial institutions, logistics and others.

**Figure 3:**
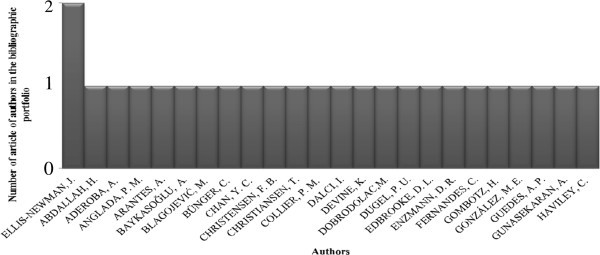
**Relevance of the authors in the bibliographic portfolio.** Source: Authors.

Another indicator used was the number of articles per author in the portfolio, as shown in Figure 3. Altogether 59 authors were identified in the portfolio; none of the authors had a higher participation. The only author with two articles in the portfolio was Ellis-Newman, J.

The keywords index found in the portfolio was also analyzed. The highlight is the keyword *activity-based costing* the search root word, followed by health services, economics, hotels. This may imply that the ABC method is used in service organizations with the intention of reducing costs and improving productivity. For articles on health, there is a reservation: they focus their application of the ABC method in restricted areas or department of a health organization.

## Bibliometric analysis of the portfolio references

**Figure 4:**
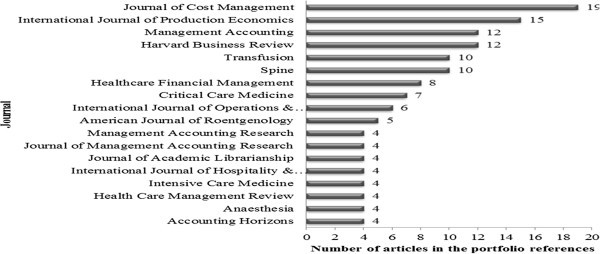
**J*ournals*relevance in the portfolio of articles.** Source: Authors.

This subsection deals with the bibliometric analysis of the articles portfolio references. In total 305 references from 21 articles were recorded, pointing out that this result considers, only journal articles. It was found that 149 titles of journals or scientific journals were cited. Regarding the most relevant journals in the portfolio references, Journal of Cost Management and International Journal of Production Economics. Figure 4 illustrates the journals that had four or more articles in the portfolio reference.

**Figure 5:**
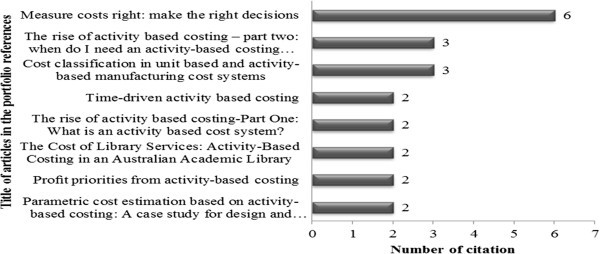
**Most cited articles in the portfolio references.** Source: Authors.

The next indicator analyzed was the most cited articles (Figure 5) in the portfolio references, for such the number of times it was cited was counted.

By means of Figure 5, it can be seen that the most cited article in the portfolio references was: Measure costs right: make the right decisions written by Robin Cooper and Robert S. Kaplan published in Harvard Business Review in 1988. And with three citations, the articles by Robin Cooper are shown: The rise of activity based costing – part two: when do I need an activity-based costing system published in the Journal of Cost Management in 1988 and, Cost classification in unit-based and activity-based manufacturing cost systems, also published in the Journal of cost Management in 1990.

**Figure 6:**
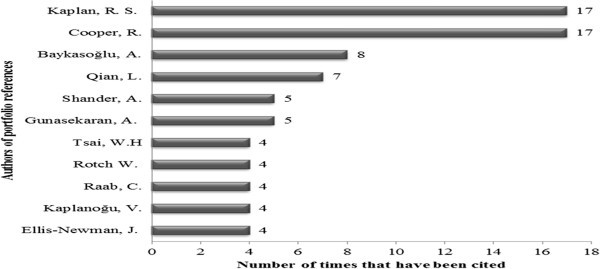
**Most cited authors in the portfolio references.** Source: Authors.

Finally, the most cited author was investigated in the portfolio references. The highlighted authors are shown in Figure 6.

So this section held bibliometric analysis of the articles portfolio references built on the application of the ABC method of costs in service.

## Analysis: portfolio versus portfolio references

**Figure 7:**
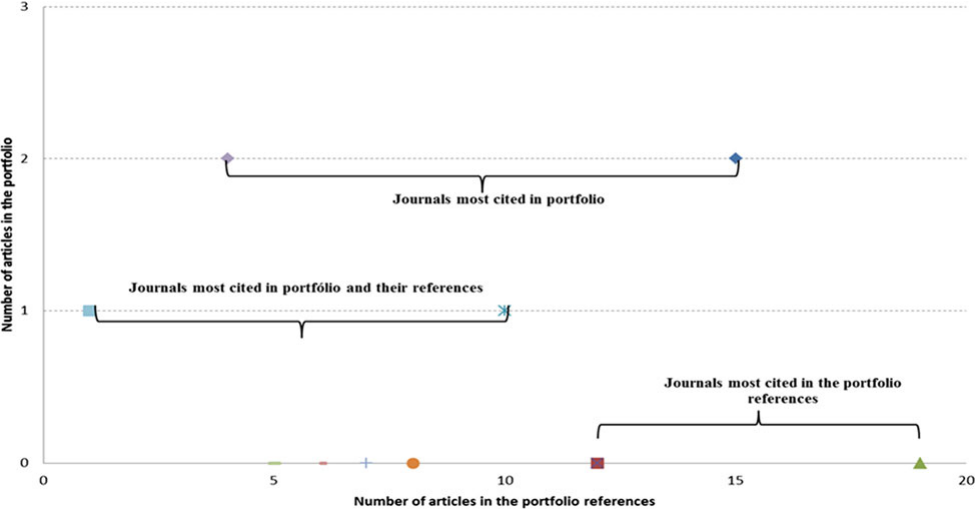
**Most prominent journals in articles portfolio and their references.** Source: Authors.

Having the portfolio built with 21 representative articles on the subject using the ABC method in services, it was identified: the most prominent journals, titles of highlighted articles; authors who have excelled. Figure 7 shows the most prominent journals in portfolio and in its references.

The most cited article in the portfolio references was the Journal of Cost Management (cited 19 times in portfolio references), but no article was accounted for in the portfolio itself with this journal. This might be due to the fact that the research topic is very specific in application in services and thus causing many articles, for example, applied in industry have been left out. The second prominent journal in the portfolio as well as in its references was the International Journal of Production Economics (cited 15 times in references, and two in the portfolio). Another prominent journal in the portfolio was Journal of Academic Librarianship presenting two citations in the portfolio and only four in its references.

**Figure 8:**
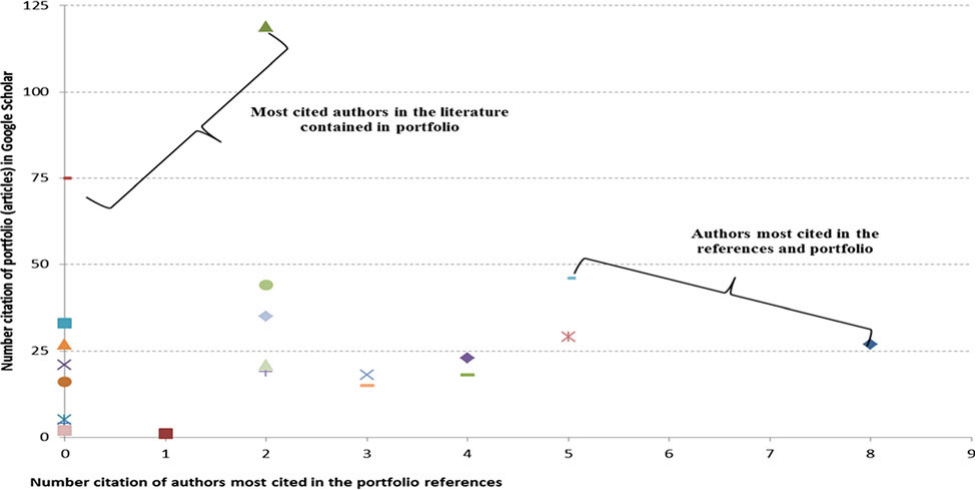
**Authors and most outstanding title in articles portfolio of and its references.** Source: Authors.

The following analysis refers to the authors featured in the portfolio and its references (Figure 8). The most cited author in the literature is Chan, Yee-Ching Lilian with article Improving hospital cost accounting with activity-based costing, published in Health Care Management Review in 1993. This article was checked in Google Scholar and had 119 citations on 13/03/2012, and this number tends to increase every day that it is checked due the fact, that this or any other article is being cited in research and studies. References in the article portfolio have been cited 2 times. The second most cited paper in the literature (75 citations in Google Scholar and not once in the portfolio references) and present the articles portfolio is A new method of accurately Identifying costs of individual Patients in intensive care: the initial results of Edbrooke, D. L.; Stevens, V. G., Hibbert, C. L., published in Intensive Care Medicine in 1997.

**Figure 9:**
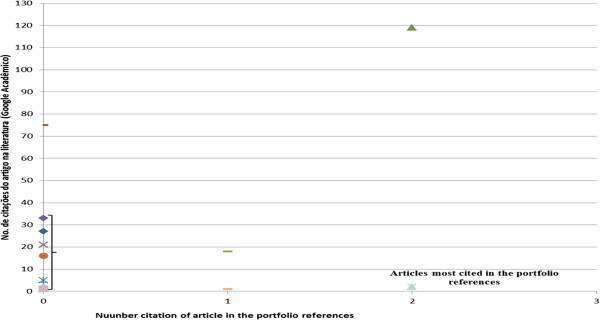
**Most cited articles in the references and that are in the portfolio.** Source: Authors.

Also in Figure 8, the most cited authors in the references were Baykasoǧlu, A. and Gunasekaran, A., with 8 and 5 citations respectively. It is important to note that the citations of these authors do not relate to those contained in the portfolio, but to other works by them. The next and final analysis relates to the most outstanding items in the portfolio and its references, as shown in Figure 9. The articles present in the portfolio and the most cited in the references are: Improving hospital cost accounting with activity-based costing (Chan, Yee-Ching Lilian); Logistic costs case study - an ABC approach (Themido, I., Arantes, A., Fernandes C., Guedes, A.P.); and Application of activity-based costing (ABC) is a Peruvian NGO healthcare provider (Waters, H., Abdallah, H.; Santillán, D.), each with two citations.

**Figure 10:**
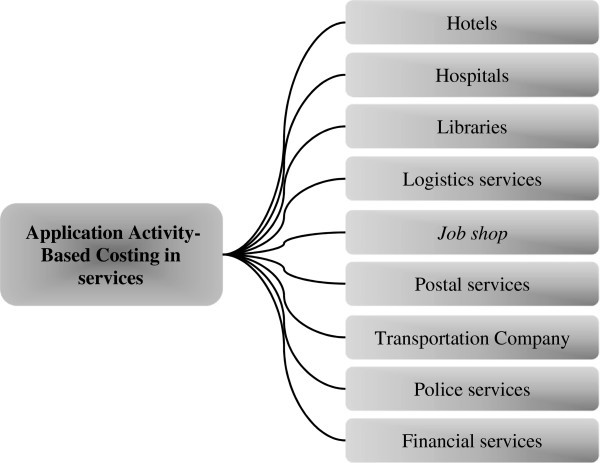
**Application of the ABC method in different types of service found in the bibliographic portfolio.** Source: Authors.

Therefore, with this research it was possible to the researcher knowledge needed to start a study on the subject application of the ABC method in services and also classify the types of services where they were applied (Figure 10).

In general, the characteristics of the built portfolio were:

- The majority (8 articles) of the applications of the ABC method is in organizations that provide health services.
- The ABC method is used in its traditional or adapted form, sometimes it is integrated into some kind of tool such as QFD and AHP.
- Most of these researches use the method to identify activities that add value and those that cause injury, in order to improve their productivity and competitiveness.

Therefore, using the ABC method of cost requires a detailed study of organizations so that they can conduct an analysis to identify activities which consume resources. An organization wishing to implement the ABC method should provide sufficient resources, but also those involved in the project should carefully observe which the cost drivers are to be used. If the organization does not provide the necessary resources, the results are disastrous.

## Conclusions

Organizations have a constant need to be prepared to continue competing, which shows the search for alternatives for their stay in the market. It has become a common goal in today's business environment, to improve efficiency and restructure organization, turning it effective. In this context, information about costs has become increasingly important to support and justify the process of decision-making. For the implementation of ABC to be successful, top management commitment will be needed, so that all objectives are in accordance with: strategy, quality and performance assessment, awareness of time that is required for this implementation and experience in media.

This article aims to build an article portfolio about the application of the ABC method in services to contribute to research on this subject. For this purpose, a three-stage procedure was performed, Planning, Implementation and Synthesis for the preparation of this portfolio. Searches carried form via journal portal Capes (in the databases *ISI Web of Knowledge* and *Scopus*), comprising the period 1990–2011.

The formation of the bibliographic portfolio on the ABC method in service firms resulted in the identification of 21 articles presented in Frame 1. A bibliometric analysis of the portfolio and its references was subsequently held. Finally a general analysis of what is each of these articles. And, by analyzing the content of each article it was possible to classify in what types of service they were applied. The majority (8 articles) of the applications of the ABC method is in organizations that provide health services.

Some limitations in the research can be highlighted: (i) only articles published in international journals were considered, (ii) research sources such as books, dissertations, theses, proceedings of conferences, events were excluded, (iii) the time period considered for the search was 1990–2011, (iv) only two databases were considered, and (v) only databases freely accessible via the portal Capes were considered. This work, besides contributes to fostering discussions in academic science, also contributes to the business environment. For the survival of small organizations depends on their ability to generate profits. However, the generation of profits does not occur randomly, it requires careful planning, several management analyzes for decision-making.

Therefore, it is important to note that a well-structured costing method according to the needs of the organization supports consistent decision-making and is an efficient management tool. An organized cost analyzes and control system, appropriate to the aims of the organization, outlines what is happening, how best to allocate resources and therefore optimize the results.
